# Microvascular characteristics of lower-grade diffuse gliomas: investigating vessel size imaging for differentiating grades and subtypes

**DOI:** 10.1007/s00330-018-5738-y

**Published:** 2018-10-01

**Authors:** Hong Guo, Houyi Kang, Haipeng Tong, Xuesong Du, Heng Liu, Yong Tan, Yizeng Yang, Sumei Wang, Weiguo Zhang

**Affiliations:** 1 Department of Radiology, Institute of Surgery Research, Daping Hospital, Army Medical University, 10# Changjiang Branch Road, Chongqing, People’s Republic of China 400024; 2 Chongqing Clinical Research Centre of Imaging and Nuclear Medicine, Chongqing, 400042 China; 3 Department of Medicine, Gastroenterology Division, University of Pennsylvania School of Medicine, Philadelphia, PA 19104 USA; 4 Department of Radiology, Division of Neuroradiology, Hospital of the University of Pennsylvania, 3400 Spruce Street, Philadelphia, PA 19104 USA

**Keywords:** Glioma, Microvessels, Molecular typing, Vessel size imaging

## Abstract

**Objectives:**

Vessel size imaging (VSI) could reveal average microvessel diameter. The aim was to investigate microvascular characteristics and the efficacy of VSI in lower-grade glioma (LGG) grading and subtype differentiation based on 2016 classification of central nervous system tumours.

**Methods:**

Fifty-seven LGG (grade II/III, 36/21) patients who received VSI examination before surgery were retrospectively analysed. The average (*R*_*mean*_) and maximum (*R*_*max*_) vessel size indexes were obtained. The long (VD_max_) and short (VD_min_) vascular diameter, microvascular area (MVA) and density (MVD) were obtained using paraffin specimens. The patients were divided into grades II and III, and histological and molecular subtypes. The differences among microvascular parameters of different subtypes and grades were compared. Two-sample *t*-test, analysis of variance test, Mann-Whitney test, the Kruskal-Wallis test and Pearson correlation analysis were used for statistics.

**Results:**

*R*_*mean*_, *R*_*max*_, VD_min_, VD_max_, and MVA were higher in grade-III than in grade-II LGGs (*p* < 0.05) in each type except the isocitrate dehydrogenase (IDH) mutant with 1p/19q-intact type. For grade II, the IDH mutant with 1p/19q co-deleted and IDH wildtype possessed more dominant angiogenesis than IDH mutant with 1p/19q-intact type, revealed by lower *R*_*mean*_, *R*_max_ and VD_min_ while higher MVD for the former (*p* < 0.05), the same as oligodendroglioma versus astrocytoma. *R*_*mean*_ and *R*_*max*_ correlated with VD_min_ (*r* = 0.804, 0.815, *p* < 0.05), VD_max_ (*r* = 0.766, 0.774, *p* < 0.05) and MVA (*r* = 0.755, 0.759, *p* < 0.05), respectively, while they had no correlation with MVD (*r* = -0.085, -0.080, *p* > 0.05).

**Conclusions:**

VSI holds great potential for non-invasively revealing microvascular characteristics of LGGs pre-surgery and differentiating their grades and molecular subtypes.

**Key Points:**

*• VSI can assist in differentiating grade-II and -III gliomas.*

*• The IDH gene and 1p/19q chromosome may influence the angiogenesis in grade-II gliomas.*

*• VSI is valuable for differentiating the molecular subtypes of grade-II gliomas.*

**Electronic supplementary material:**

The online version of this article (10.1007/s00330-018-5738-y) contains supplementary material, which is available to authorized users.

## Introduction

The biological characteristics of lower-grade gliomas (LGGs, grades II and III) are diverse [1, 2]. Characterised by higher chemotherapy sensitivity and better prognosis, oligodendroglioma has a more indolent clinical course than astrocytoma. Influenced by inter/intra-observer reproducibility in defining cell lineage and grade, and sample errors, histological diagnosis has inevitable limitations. The classification of LGGs, based on the mutational status of isocitrate dehydrogenase (IDH) and deletion of the 1p/19q chromosome, could reflect the genetic information of subtypes and predict prognosis. It is superior to the histological classification [2]. The 2016 classification criteria classify LGGs into three categories [3]. The vast majority of astrocytomas are classified as the IDH mutant with 1p/19q-intact (IDH^MUT^/1p/19q^+^) type, while oligodendrogliomas are classified as the IDH mutant with 1p/19q co-deletion (IDH^MUT^/1p/19q^−^) type. Oligoastrocytomas are divided into IDH^MUT^/1p/19q^+^ or IDH^MUT^/1p/19q^−^ type objectively. The IDH^MUT^/1p/19q^−^ type is sensitive to radiotherapy and chemotherapy for the 1p/19q co-deletion. The median survival time for the IDH^MUT^/1p/19q^−^ type is 8 years, while that for the IDH^MUT^/1p/19q^+^ type is 6.3 years [2]. Patients with IDH wildtype (IDH^WT^), which presents telomerase reverse transcriptase mutation and epidermal growth factor receptor amplification, experience poor prognosis with a median survival time of only 1.23 years [4]. Although there is no difference in the prognosis between grade II and III for the IDH^MUT^/1p/19q^+^ type, traditional grading remains a significant outcome predictor and provides additional prognostic value among the molecular subsets, particularly for the IDH^WT^ type [2, 5]. Therefore, an accurate pre-surgical diagnosis of LGGs, the molecular subtype is particularly significant in guiding the clinical management and determining the prognosis.

Microvascular proliferation characteristics are critical for grading and subtype of LGGs [6]. The oligodendroglioma often has unique vascularity manifesting as “chicken-wire” vasculature. However, the astrocytoma displays similar features compared with normal brain [7]. As the grade increased, vascular angiogenesis is more prevalent, and vascular morphology is more distorted with dilated lumen. In addition, the status of the IDH gene and the 1p/19q chromosome can affect the extent of angiogenesis in diffuse gliomas [8–10]. The relative cerebral blood volume (rCBV) of the IDH wildtype has been proved to be higher than that of the IDH mutant type in LGGs [8]. The rCBV of the 1p/19q co-deleted type was higher than 1p/19q intact type in oligodendroglioma [9]. However, it remains controversial when assessing oligodendroglioma progression and grading using rCBV, due to fact that the “chicken-wire” vasculature often leads to hyperperfusion [11–14]. Furthermore, the rCBV contains both microvascular density and diameter information, which could hardly reflect the microvascular characteristics precisely.

Vessel size imaging (VSI) is an emerging magnetic resonance imaging (MRI) technique that can accurately reveal microvessels with an average diameter of 2–50 μm [15–18]. It is based on the difference in the contribution of small vessels to the transverse relaxation rates of gradient echo and spin echo sequences [19]. The transverse relaxation changes before and after injecting contrast agents are positively correlated with the microvascular diameters. The vessel size index, *R*, can be obtained by using functions that correlate with the susceptibility and apparent diffusion coefficient [20, 21]. Previous VSI studies mainly focused on the diagnosis of grade-II to -IV gliomas. Studies concerning integrating IDH genetic and 1p/19q chromosome information with microvessels were performed mainly by dynamic susceptibility-weighted MRI, while VSI was seldom introduced. In this study, we analysed the microvascular characteristics of LGGs according the 2016 classification for central nervous system tumours and investigated the value of VSI in pre-surgical LGG grading and subtype differentiation.

## Materials and methods

### Patients

This retrospective study was approved by the institutional review board of our hospital, and the informed consents were obtained. Fifty-seven patients (42.58 ± 12.45 years) were collected in our hospital from June 2013 to May 2016. The patients were confirmed grade-II or -III diffuse gliomas by pathological examination after surgery. Any patient received no treatment before the operation, and all patients underwent surgery within 2 weeks after MRI examination.

### MR image acquisition

The VSI sequence was provided by GE Healthcare (Chicago, IL, USA) and installed in 1.5-T MR scanner (Signa HDX; GE Healthcare). To ensure the image quality and accurate diagnosis, patients received conventional MR examination using 3.0-T MR scanner (Verio; Siemens, Erlangen, Germany), and the cases with suspected LGGs on 3.0-T MRI were recruited to undergo VSI scanning. All cases were performed on the anterior commissure-posterior commissure line or on its parallel lines. The conventional MRI scanning parameters were as follows: T1WI (TR/TE = 250/2.67 ms), T2WI (TR/TE = 4,900/100 ms), T2-FLAIR (TR/TE = 8,000/94 ms), matrix = 320 × 320, and slice thickness = 5 mm. The gradient echo–spin echo–echo planar imaging sequence was employed for VSI scanning. The tumour slice with the largest area was selected as the scan centre plane, and seven slices were collected at 50 frames per slice. In the second frame, a high-pressure injector (REFXD; Ulrich, Ulm, Germany) was utilised for an intra-antecubital vein bolus injection of Gd-DTPA (Consun Pharmaceutical Group, Guangzhou, China), with the dose of 0.2-0.3 mmoL/kg body weight at a speed of 3.0 mL/s. Equal volume of saline was then injected to rinse the tube. The scanning parameters for VSI were as follows: field of view = 24 cm × 24 cm, TR = 1,500 ms, TE (GE = 30 ms, SE = 100 ms), flip angle = 90°, matrix size = 64 × 64 and slice thickness = 5 mm.

### MR image analysis

The VSI data were imported to VSI post-processing software in GE Advantage 4.9 Workstation. The largest area plane of the tumour and the contra lateral normal brain tissue on the same slice were selected as the input values. The time-signal intensity curve was obtained, and *R* was calculated using the following equation:

$$ R=0.425\times {\left(\frac{ADC}{\gamma \times \triangle \chi \times {B}_0}\right)}^{1/2}{\left(\frac{\triangle {R_2}^{\ast }}{\triangle {R}_2}\right)}^{3/2} $$

*R* is the vessel size index, ADC is the apparent diffusion coefficient, which is 0.8 μm^2^/ms, γ is the gyromagnetic ratio of a hydrogen proton, which is 42.58 MHz, △χ is the increase in paramagnetic susceptibility, B_0_ is the main magnetic field strength (1.5 T), and △R_2_* and △R_2_ are the changes in the transverse relaxation of GE and SE sequences when the time-signal curve starts to drop to its minimum, respectively.

The threshold of the VSI maps was adjusted to 0-120 μm. The hot-spot method was used by two experienced neuroradiologists (who had 10 and 23 years of experience, respectively) who were blinded to IDH mutation and 1p/19q co-deleted status when analysing the VSI maps. At least five circular regions of interest with the same size of 56 mm^2^ were selected in the solid component of the tumour, avoiding large vessels, cystic, necrotic, haemorrhagic components and the leptomeningeal region of the tumour by referencing conventional MR images (Supplementary Fig. S1) [22]. The values measured in the two regions of interests and ten regions of interests with maximum values by the two observers were then averaged to represent the maximum vessel size index (*R*_*max*_) and mean vessel size index (*R*_*mean*_) respectively, for each tumour.

### IDH1/2 analysis

Genomic DNA was isolated from paraffin-embedded tumour tissues using the QIAmp DNA mini-kit (Qiagen, Valencia, CA, USA). DNA sequencing was performed by Chongqing Maobai Technology Incorporation. IDH1 and IDH2 alterations of the mutational hot-spot codons R132 and R172 were determined via polymerase chain reaction. The primers used for IDH were as follows: IDH1 (sense primer: 5′-CGGTCTTCAGAGAAGCCATT-3′, antisense primer: 5′- GCAAAATCACATTATTGCCAAC-3′) and IDH2 (sense primer: 5′-TCTTCCGGGAGCCCATCAT-3′, antisense primer: 5′-CTTCCCACTCCTTGACACCAC-3′).

### 1p/19q co-deleted

Fluorescence in situ hybridisation was performed for each tumour to detect 1p/19q co-deletion (Vysis 1p36/1q25 and 19q13/19p13 FISH Probe Kit; Abbott Molecular, De Plaines, IL, USA). For each probe, at least 100 non-overlapping nuclei were selected. When the proportion of missing nuclei was over 30%, the sample was defined as exhibiting chromosomal loss.

### Pathological analysis

The paraffin-embedded tumour tissues were sliced into 4-μm sections for CD34 staining. Olympus BX41 microscope was utilised for section observation. The hot-spot approach was used, in which the entire section was scanned with a low-power lens (40×, 1.308 × 1.757 mm^2^/view). Five hot spots were then selected using a high-power lens (200×) for image collection [23]. Cystic lesion, necrosis, and non-tumour regions were also disregarded. Image-Pro Plus 6.0 (Media Cybernetics, Rockville, MD, USA) was utilised to measure the short diameter (VD_min_), long diameter (VD_max_), microvascular area (MVA), and microvascular density (MVD).

### Statistical analysis

All values were expressed as mean ± standard deviation. The inter-observer agreement between the two observers was analysed by the intra-class correlation coefficient. The two-sample *t*-test or analysis of variance test was employed when comparing between histological groups in which data of certain groups were proved to be normal distribution and homogeneity of variance. Mann-Whitney test or the Kruskal-Wallis and Nemenyi was applied in molecular groups for uniformity of variances. Pearson correlation analysis was performed between VSI indexes and microvascular features. Receiver operating characteristic curves were applied to evaluate the value of parameters in differentiating grades and subtypes. Statistical analysis was performed using commercially available software packages (SPSS 22.0 and Prism Graphpad 6.0 verison). *p* < 0.05 was considered to be statistically significant.

## Results

### Patients

The clinical characteristics of the 57 cases are summarised in Table 1. The histological classification included astrocytoma, oligodendroglioma and oligoastrocytoma. There were 40 cases of IDH mutant (grade II/III, 28/12), 17 cases of IDH wildtype (grade II/III, 8/9), 25 cases of 1p/19q intact (grade II/III, 16/9) and 32 cases of 1p/19q co-deleted (grade II/III, 20/12). Combining the IDH gene and the 1p/19q chromosome, the tumours were divided into the IDH^MUT^/1p/19q^+^ (13 cases), IDH^MUT^/1p/19q^−^ (27 cases) and IDH^WT^ (17 cases) type.

**Table 1:** Patient demographics and clinical characteristics

	Type ^a^	IDH^MUT^/1p/19q^+^		IDH^MUT^/1p/19q^−^			IDH^WT^
	Grade	II	III	II	III	II	III
	Total	12	1	16	11	8	9
Sex	Male (%)	5 (41.7)	1 (100)	6 (37.5)	8 (72.7)	2 (25.0)	6 (33.3)
	Female (%)	7 (58.3)	0 (0)	10 (62.5)	3 (27.3)	6 (75.5)	3 (66.7)
Histology	Astrocytoma (%)	11 (91.7)	0 (0)	0 (0)	0 (0)	3 (37.5)	6 (66.7)
	Oligodendroglioma (%)	0 (0.0)	0 (0)	9 (56.3)	7 (63.3)	4 (50.0)	2 (22.2)
	Oligoastrocytoma (%)	1 (8.3)	1 (100)	7 (43.7)	4 (36.7)	1 (12.5)	1 (11.1)

^a^IDH^MUT^/1p/19q^+^ IDH mutant with 1p/19q intact type, IDH^MUT^/1p/19q^−^ IDH mutant with 1p/19q co-deleted type, IDH^WT^ IDH wildtype

### VSI and microvascular characteristics of histological subtypes

**Fig. 1:**
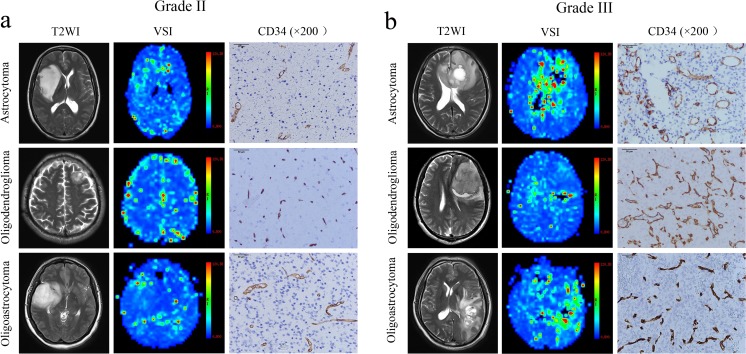
Microvascular characteristics of different histological subtypes of lower-grade gliomas. **a** Microvascular characteristics of different histological subtypes in grade-II gliomas. All the subtypes showed low perfusion on vessel size imaging maps, and the perfusions of astrocytoma (woman, 42 years) and oligoastrocytoma (woman, 34 years) are slightly higher compared with that of oligodendroglioma (man, 50 years). CD34 staining results demonstrated that the angiogenesis of oligodendroglioma is more exuberant than that of astrocytoma, evidenced by higher microvascular density and smaller diameter for the former. **b** Microvascular characteristics of different histological subtypes in grade-III gliomas. All the subtypes showed high perfusion on vessel size imaging maps and exuberant angiogenesis on CD34 stained sections

**Fig. 2:**
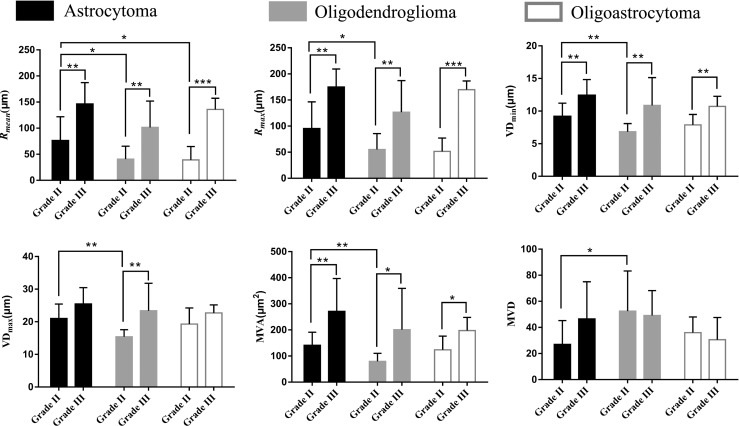
Histograms of average vessel size index (*R*_*mean*_), and maximum vessel size index (*R*_*max*_), short vascular diameter (VD_min_), long vascular diameter (VD_max_), microvascular area (MVA) and microvascular density (MVD) for comparison of grades and histological subtypes. *p < 0.05, **p < 0.01, ***p < 0.001

The averages of the microvascular parameters for each histological subtype are presented in Supplementary Table S1. The grade-III gliomas showed higher perfusion compared with that of grade II on VSI maps for each subtype (Figs. 1a and 2b). The *R*_*mean*_, *R*_*max*_, VD_min_ and MVA values in grade III were higher than those of grade II (*p <* 0.05) in aspects of all histological subtypes (Fig. 2). The difference of VD_max_ between grade II and III was only observed in oligodendroglioma patients (*p =* 0.004). The MVD did not differ between grades II and III in any histological subtype (*p >* 0.05). For grade-II gliomas, all parameters were different between astrocytoma and oligodendroglioma (*p* < 0.05). There was only *R*_*mean*_ difference between astrocytoma and oligoastrocytoma (*p* = 0.003). There was no difference in any parameter between oligodendroglioma and oligoastrocytoma, among subtypes in grade III (*p >* 0.05).

### VSI and microvascular characteristics of molecular subtypes

**Fig. 3:**
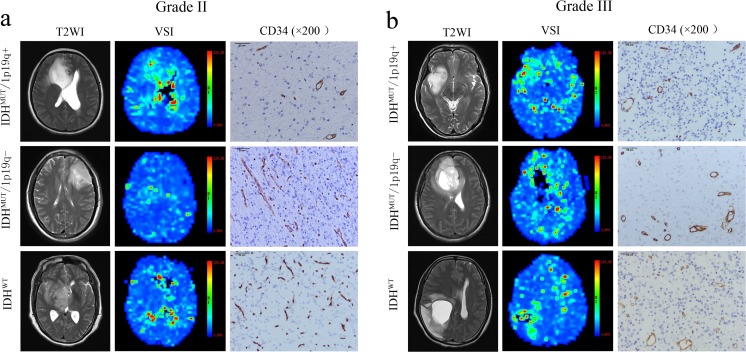
Microvascular characteristics of different molecular subtypes of lower-grade gliomas. **a** Microvascular characteristics of different molecular subtypes in grade-II gliomas. All the subtypes showed low perfusion on vessel size imaging maps, and the perfusion of the IDH-mutant with 1p/19q-intact (IDH^MUT^/1p/19q^+^) type (male, 48 years) was higher than that of the IDH-mutant with 1p/19q-codeleted (IDH^MUT^/1p/19q^−^) type (female, 46 years) and the IDH-wild (IDH^WT^) type (female, 40 years). CD34 staining results demonstrated that the angiogenesis of the IDH^MUT^/1p/19q^−^ and IDH^WT^ type are more exuberant than that of the IDH^MUT^/1p/19q^+^ type, evidenced by higher microvascular density and smaller diameter for the former. **b** Microvascular characteristics of different molecular subtypes in grade-III gliomas. All the subtypes showed high perfusion on vessel size imaging maps and exuberant angiogenesis on CD34 stained sections

**Fig. 4:**
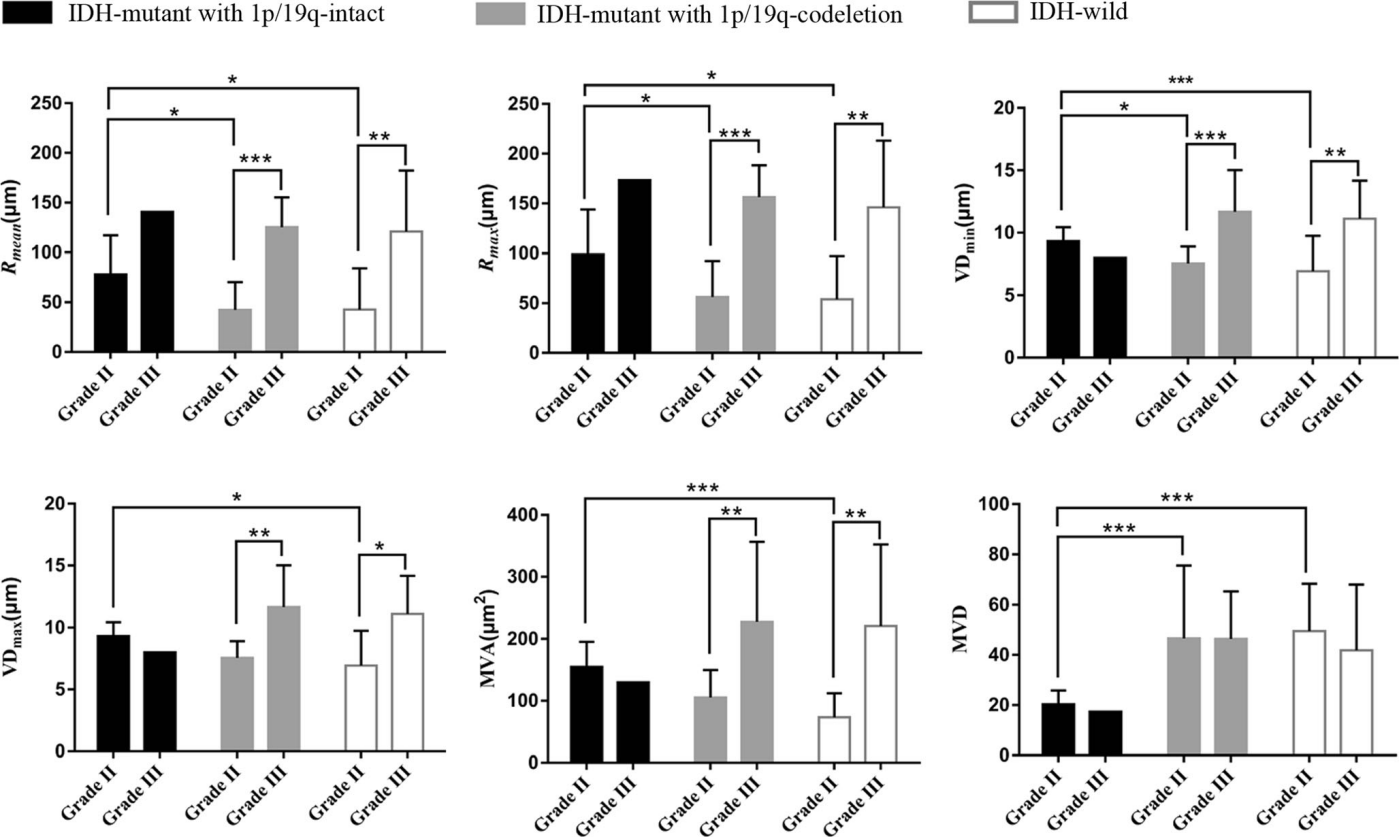
Histogram of average vessel size index (*R*_*mean*_), and maximum vessel size index (*R*_*max*_), short vascular diameter (VD_min_), long vascular diameter (VD_max_), microvascular area (MVA) and microvascular density (MVD) for comparison of grades and molecular subtypes. *p < 0.05, **p < 0.01, ***p < 0.001

The patients were divided into the IDH^MUT^/1p/19q^+^, IDH^MUT^/1p/19q^−^ and IDH^WT^ subtype. Within each subtype, a comparison between grade II and III was performed (Supplementary Table S2). The grade-III gliomas showed higher perfusion compared with that of grade II on VSI maps for each subtype (Fig. 3a, b). As there was only one case of the IDH^MUT^/1p/19q^+^ type in grade-III gliomas, the IDH^MUT^/1p/19q^+^ type was not analysed. *R*_*mean*_, *R*_*max*_, VD_min_, VD_max_ and MVA in grade III were higher than those in grade II (*p* < 0.05) in IDH^MUT^/1p/19q^−^ and IDH^WT^ types, while MVD showed no significant difference in the two groups (*p* > 0.05; Fig. 4). For grade-II gliomas, the values of *R*_*mean*_, *R*_*max*_, VD_min_ of IDH^MUT^/1p/19q^+^ type were higher compared with the IDH^MUT^/1p/19q^−^ type (*p* < 0.05), while MVD was lower (*p* = 0.000). For grade-II gliomas, the values of *R*_*mean*_, *R*_*max*_, VD_min_, VD_max_, and MVA of IDH^MUT^/1p/19q^+^ type were higher compared with the IDH^WT^ type (*p* < 0.05), while MVD was lower (*p* = 0.000). However, no difference was observed between IDH^MUT^/1p/19q^−^ and IDH^WT^ type in grade II, among subtypes in grade III (*p* > 0.05; Fig. 4).

### Receiver operating characteristic curves analysis

**Fig. 5:**
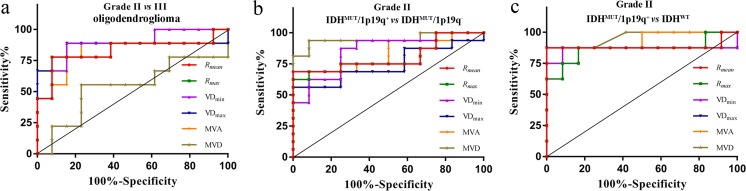
Receiver operating characteristic curves for differentiating grade-II and grade-III gliomas in oligodendroglioma (**a**), IDH mutant with 1p/19q intact (IDH^MUT^/1p/19q^+^) and IDH mutant with 1p/19q co-deleted (IDH^MUT^/1p/19q^−^) type in grade-II gliomas (**b**), IDH^MUT^/1p/19q^+^ and IDH wildtype (IDH^WT^) in grade-II gliomas (**c**)

In differentiating grade-II from grade-III gliomas, the accuracy of *R*_*mean*_, *R*_*max*_, and VD_min_ and MVA were all higher than 0.8 (*p <* 0.05, Supplementary Fig. S2). The area under the curve (AUC) of VD_max_ was higher than 0.8 when differentiating grade-II from grade-III gliomas both in oligodendroglioma and IDH^MUT^/1p/19q^−^ type (*p <* 0.05). The AUC of MVD was not significant in differentiating grade-II from -III gliomas (*p >* 0.05), but it was higher when differentiating the astrocytoma and oligodendroglioma, IDH^MUT^/1p/19q^+^ and IDH^MUT^/1p/19q^−^, IDH^MUT^/1p/19q^+^ and IDH^WT^ in grade II (AUC = 0.870, 0.948, 0.948, respectively; *p < *0.05). *R*_*mean*_, *R*_*max*_, VD_min_, VD_max_ and MVA could also differentiate astrocytoma versus oligodendroglioma, IDH^MUT^/1p/19q^+^ versus IDH^WT^ in grade-II gliomas. In differentiating IDH^MUT^/1p/19q^+^ and IDH^WT^ in grade-II gliomas, the accuracy of *R*_*mean*_, *R*_*max*_, VD_min_, VD_max_ and MVA was significant (AUC = 0.885, 0.865, 0.865, 0.875, 0.938, respectively; *p <* 0.05) (Fig. 5, Supplementary Table S3 and Supplementary Fig. S2).

### Correlation analysis

**Fig. 6:**
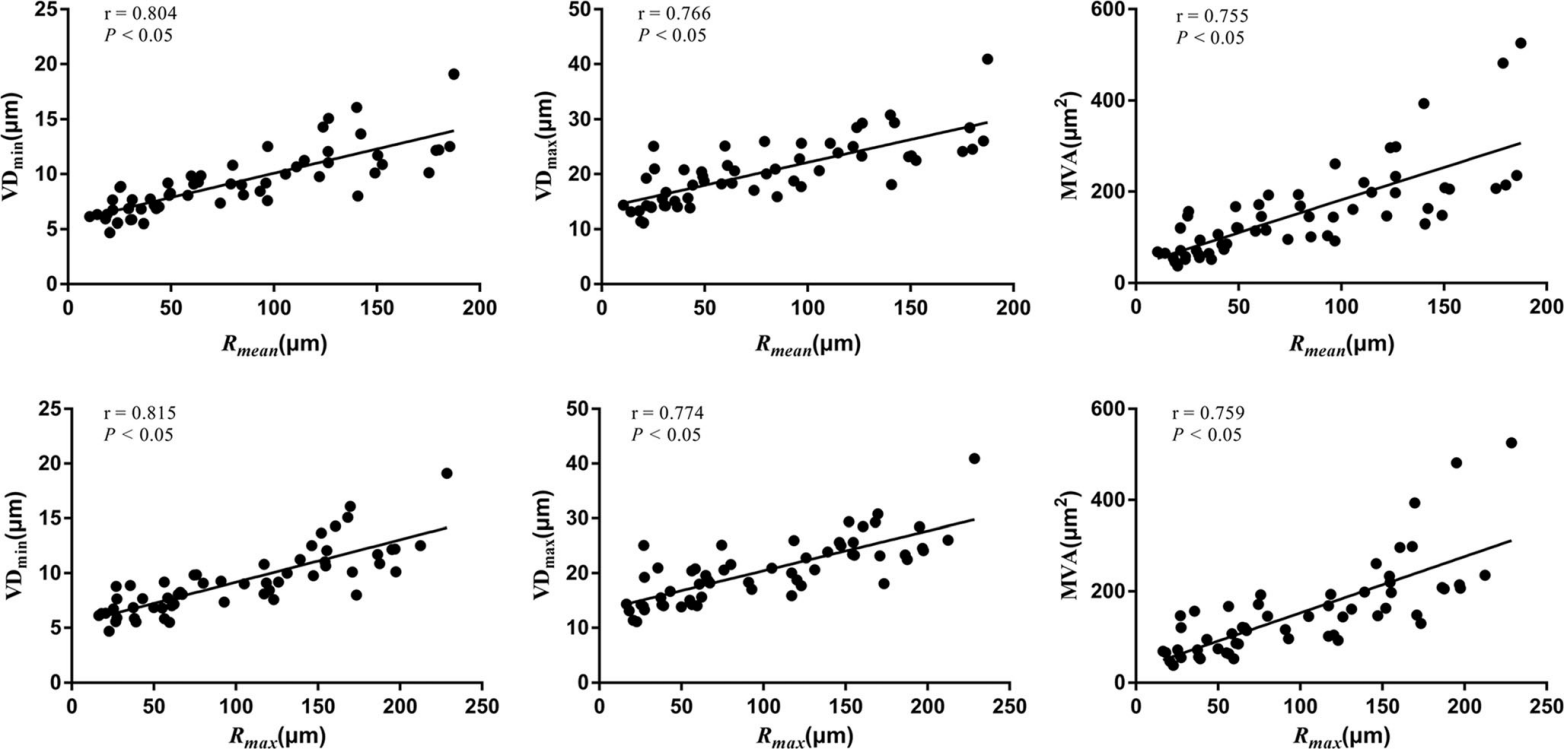
Correlation between vessel size imaging parameters and pathological indices. The short vascular diameter (VD_min_), long vascular diameter (VD_max_) and microvascular area (MVA) are positively correlated with average vessel size index (*R*_*mean*_) and maximum vessel size index (*R*_*max*_), respectively

There was good positive correlation between *R*_*mean*_ and VD_min_, VD_max_ and MVA (*r* = 0.804, 0.766, 0.755, respectively; *p* < 0.05, Fig. 6). A closed positive correlation was observed between *R*_*max*_ and VD_min_, VD_max_ and MVA (*r* = 0.815, 0.774, 0.759, respectively; *p* < 0.05). There was no correlation between MVD and *R*_*mean*_, *R*_*max*_ (*r* = -0.085, -0.080, *p* = 0.529, 0.552, respectively; Supplementary Fig. S3).

### Inter-observer agreement

For all the 57 cases, the inter-observer consistency was adequate for *R*_*mean*_ and *R*_*max*_. The intra-class correlation coefficient value for *R*_*mean*_ was 0.973 (95% confidence interval: 0.954, 0.984, *p* < 0.05) and that of *R*_*max*_ was 0.957 (95% confidence interval: 0.929, 0.975, *p* < 0.05).

## Discussion

In this study, we found that regardless of histological or molecular subtypes, vascular diameter could serve as a good index to reflect the microvascular proliferation between grade-II and -III gliomas, except for the IDH^MUT^/1p/19q^+^ type. Different microvascular characteristics among different subtypes were only observed in grade-II gliomas, and vascular diameter and MVD could serve as efficient indices for differentiating oligodendroglioma and astrocytoma, IDH^MUT^/1p/19q^+^ and IDH^MUT^/1p/19q^−^ type, IDH^MUT^/1p/19q^+^ and IDH^WT^ type. VSI could directly reflect the microvascular diameter, evading the interference of MVD. It could non-invasively identify various subtypes in grade-II gliomas, which further extends the clinical application value of VSI.

The grade-III gliomas possess more exuberant vascularisation than grade-II gliomas [24]. This study showed that vascular diameter and MVA were different between grade II and III in all subtypes, except for the IDH^MUT^/1p/19q^+^ type. VSI could be a reliable method for LGG grading. However, there was no difference in the MVD between different grades of LGGs, as MVD was not a reliable indicator of the progress and malignancy of gliomas [23]. Glioma grading has been confronted with challenges. Some studies found that the prognosis was similar between grade-II and -III gliomas for the IDH^MUT^/1p/19q^+^ type but different for the IDH^WT^ type [4, 5]. It is worth discussing whether it is reasonable for glioma grading according to the traditional histological characteristics.

Conventional MRI has limitations in differentiating the histological subtypes of LGGs. In general, oligodendroglioma is involved the cortex more frequently than astrocytoma, and showed more calcification, but conventional MR was subjective. Cystic degeneration, haemorrhage and enhancement were observed in some oligodendrogliomas, similar with high-grade gliomas [25]. Although rCBV is significant in determining astrocytoma and oligodendroglioma in grade-II gliomas, grade-II oligodendroglioma may be misdiagnosed in higher grade due to the “chicken-wire” network [26]. In this study, vascularisation of oligodendroglioma is more exuberant than that of astrocytoma in grade-II gliomas, characterised by increased MVD and decreased vascular diameter. This can be explained in that oligodendroglioma is most likely to be involved in the cortex [7]. VSI can differentiate oligodendroglioma from astrocytoma in grade-II gliomas. The diameter of oligoastrocytoma microvessels has a great overlap with astrocytoma and oligodendroglioma, and VSI cannot distinguish oligoastrocytoma from astrocytoma and oligodendroglioma.

The value of rCBV of the IDH wildtype was higher than that of the IDH mutant type, and some studies found that rCBV could not differentiate IDH^MUT^/1p/19q^+^ and IDH^MUT^/1p/19q^−^ type [25, 27]. We found that vascular diameter and MVD could serve as potent indices to identify IDH^MUT^/1p/19q^+^ and IDH^MUT^/1p/19q^−^ type, IDH^MUT^/1p/19q^+^ and IDH^WT^ type in grade-II gliomas. Vascularisation was more prominent in the IDH^WT^ type than that in the IDH^MUT^/1p/19q^+^ type in grade II, characterised by higher MVD and smaller lumen for the former. The mutation of the IDH gene results in the conversion of α-ketoglutarate into 2-hydroxyglutaric acid, which inhibits tumour angiogenesis by inhibiting hypoxia inducible factor 1α [28, 29]. The IDH wildtype overexpressed hypoxia- and angiogenesis-related genes compared with the IDH mutant type, such as vascular endothelial growth factor A and angiopoietin-2 [8]. IDH mutation affects an early event in gliomagenesis that may influence the early stage of angiogenesis. At this stage, angiogenesis mainly manifested as a sprouting and highly branched vessel network, leading to smaller lumen and higher MVD [30]. The angiogenesis of the IDH^MUT^/1p/19q^−^ type was more prominent than that of the IDH^MUT^/1p/19q^+^ type in grade II. The 1p/19q co-deletion in the IDH^MUT^/1p/19q^−^ type was considered to be correlated with the “﻿chicken-wire” vasculature [31]. However, regardless of the histological or molecular subtypes, a difference between the subtypes was not apparent in grade-III gliomas. This may be related to the exuberant vascularisation in aspects of all subtypes in grade-III gliomas [32].

Vascular diameter could serve as a reliable index for differentiating the grade and molecular subtypes of LGGs. However, the quantitation of tumour microvascular diameter is time-consuming and non-routine in clinical practice. There was a fine correlation between vessel size index and vascular diameter, and VSI was a reliable imaging technique for preoperative diagnosis of LGG grade and molecular subtype in grade-II gliomas. In this study, vessel size index was not proportional to MVD. This was not in accordance with previous reports [23]. It might be related to the heterogeneity in microvascular characteristics in different subtypes.

This study has some limitations. Firstly, the IDH wildtype gliomas are not a homogenous group of tumours [4, 33]. The patients having telomerase reverse transcriptase mutation, epidermal growth factor receptor amplification which are similar to glioblastoma, experience poor prognosis. Some scholars have questioned whether there is an IDH wildtype diffusion astrocytoma [33]. Further detailed gene detection was needed in the IDH^WT^ type. In addition, the sub-classifications of oligodendroglioma and oligoastrocytoma are still indistinct. When the genotype is IDH wildtype with 1p/19q co-deletion, the tumour is classified as “not otherwise specified” [3]. The relationship between this type and IDH-wildtype glioblastoma is still unclear. Besides, as mentioned above, grading of gliomas needs further study. Furthermore, the sample size was relatively small. Finally, the VSI Calculation model was based on normal blood vessels in the brain and it only reflects vessels which the contrast agent can flow. To minimise the error, we selected regions with clear lumens when measuring blood vessels and measured all vessels in the field of view.

In summary, the microvascular characteristics of LGGs were diverse in aspects of different grades and subtypes. VSI can directly reflect the vascular diameter in LGGs and provides World Health Organization grading to some extent, as well as histological and molecular information. It could serve as a useful tool in the pre-surgical diagnosis of LGGs.

## Electronic supplementary material

ESM 1 (DOCX 9977 kb)

## Abbreviations

IDH: Isocitrate dehydrogenase
IDH^MUT^/1p/19q^+^: IDH mutant with 1p/19q intact
IDH^MUT^/1p/19q^−^: IDH mutant with 1p/19q co-deleted
IDH^WT^: IDH wildtype
LGG: Lower-grade glioma
MVA: Microvascular area
MVD: Microvascular density
*R*: Vessel size index
rCBV: Relative cerebral blood volume
VSI: Vessel size imaging
