# Supplementary material for: Microvascular characteristics of lower-grade diffuse gliomas: investigating vessel size imaging for differentiating grades and subtypes
Source: Eur Radiol. 2018 Oct 1;29(4):1893–902. doi: 10.1007/s00330-018-5738-y (PMC6420610; doi:10.1007/s00330-018-5738-y)
Supplement: Supplementary file 1 — (DOCX 9977 kb) [file 330_2018_5738_MOESM1_ESM.docx]

**Supplementary information**

**Supplementary Table 1. Measurements in histological subtypes.**

| **Index** | **Grade** | **Total** | **Astrocytoma** | **Oligodendroglioma** | **Oligoastrocytoma** |  |
| --- | --- | --- | --- | --- | --- | --- |
| ***R_mean_*** | Ⅱ | 54.32±38.04 | 76.57±45.36 | 40.76±24.66 | 39.31±25.66 |  |
|  | Ⅲ | 124.3±44.45 | 146.6±40.87 | 101.5±50.48 | 136.0±21.41 |  |
|  | *P* | **0.000** | **0.004** | **0.001** | **0.000** |  |
| ***R_max_*** | Ⅱ | 70.01±44.76 | 95.54±50.98 | 55.38±30.35 | 51.47±35.86 |  |
|  | Ⅲ | 152.9±48.59 | 175.1±34.28 | 126.8±60.34 | 169.8±16.77 |  |
|  | *P* | **0.000** | **0.003** | **0.002** | **0.000** |  |
| **VD_min_** | Ⅱ | 8.008±1.931 | 9.169±2.048 | 6.840±1.248 | 7.887±1.604 |  |
|  | Ⅲ | 11.27±3.155 | 12.41±2.425 | 10.87±4.280 | 10.72±1.559 |  |
|  | *P* | **0.000** | **0.007** | **0.004** | **0.005** |  |
| **VD_max_** | Ⅱ | 18.57±4.551 | 21.03±4.410 | 15.41±2.187 | 19.32±4.933 |  |
|  | Ⅲ | 23.79±6.123 | 25.47±5.010 | 23.39±8.427 | 22.71±2.488 |  |
|  | *P* | **0.001** | 0.063 | **0.004** | 0.147 |  |
| **MVA** | Ⅱ | 114.8±51.42 | 141.4±50.15 | 79.84±30.73 | 124.1±52.39 |  |
|  | Ⅲ | 220.4±125.1 | 271.6±125.6 | 201.2±158.0 | 197.9±50.17 |  |
|  | *P* | **0.000** | **0.003** | **0.013** | **0.018** |  |
| **MVD** | Ⅱ | 38.48±24.69 | 27.01±18.24 | 52.54±30.79 | 36.00±12.07 |  |
|  | Ⅲ | 43.14±22.11 | 46.53±28.54 | 49.21±19.09 | 30.63±17.02 |  |
|  | *P* | 0.479 | 0.081 | 0.777 | 0.485 |  |

**Supplementary Table 2. Measurements in molecular subtypes.**

| **Index** | **Grade** | **IDH^MUT^/1p/19q^+^** | **IDH^MUT^/1p/19q^−^** | **IDH^WT^** |
| --- | --- | --- | --- | --- |
| ***R_mean_*** | Ⅱ | 78.07±39.03 | 42.30±27.98 | 42.76±41.42 |
|  | Ⅲ | 140.71 | 125.4±29.99 | 121.0±61.37 |
|  | *P* | NA | **0.000** | **0.008** |
| ***R_max_*** | Ⅱ | 98.93±45.17 | 56.29±36.06 | 54.10±43.26 |
|  | Ⅲ | 173.42 | 156.4±32.12 | 146.2±67.02 |
|  | *P* | NA | **0.000** | **0.005** |
| **VD_min_** | Ⅱ | 9.324±1.124 | 7.557±1.348 | 6.934±2.830 |
|  | Ⅲ | 8.010 | 11.67±3.355 | 11.13±3.041 |
|  | *P* | NA | **0.000** | **0.010** |
| **VD_max_** | Ⅱ | 21.41±2.869 | 17.74±4.120 | 15.97±5.580 |
|  | Ⅲ | 18.11 | 24.83±6.331 | 23.15±6.137 |
|  | *P* | NA | **0.002** | **0.024** |
| **MVA** | Ⅱ | 154.9±40.67 | 105.6±44.34 | 73.37±38.76 |
|  | Ⅲ | 129.88 | 227.8±129.1 | 221.3±131.2 |
|  | *P* | NA | **0.002** | **0.008** |
| **MVD** | Ⅱ | 20.28±5.574 | 46.61±28.89 | 49.50±18.76 |
|  | Ⅲ | 17.40 | 46.49±18.86 | 41.90±26.03 |
|  | *P* | NA | 0.990 | 0.505 |

Note： IDH^MUT^/1p/19q^+^ IDH-mutant with 1p/19q-intact type, IDH^MUT^/1p/19q^−^ IDH-mutant with 1p/19q-codeleted type, IDH^WT^ IDH-wild type, NA Not Applicable

**Supplementary Table 3. Receiver operating characteristic curve analysis in distinguishing grades and subtypes.**

| **Groups** | **Index** | ***R_mean_*** | ***R_max_*** | **VD_min_** | **VD_max_** | **MVA** | **MVD** |
| --- | --- | --- | --- | --- | --- | --- | --- |
| **Astrocytoma**  **Ⅱ *vs* Ⅲ** | Cut-off | 79.94 | 154.7 | 10.82 | 25.64 | 207.02 | 23.00 |
|  | Sensitivity (%) | 71.43 | 92.86 | 92.86 | 92.86 | 92.86 | 71.23 |
|  | Specificity(%) | 100.0 | 83.33 | 83.33 | 66.67 | 83.33 | 100.0 |
|  | AUC | 0.905 | 0.905 | 0.857 | 0.762 | 0.857 | 0.857 |
| **Oligodendroglioma**  **Ⅱ *vs* Ⅲ** | Cut-off | 73.97 | 92.87 | 7.360 | 17.06 | 84.81 | 53.80 |
|  | Sensitivity (%) | 92.31 | 92.31 | 84.62 | 84.62 | 76.92 | 76.92 |
|  | Specificity(%) | 77.78 | 77.78 | 88.89 | 88.89 | 88.89 | 55.56 |
|  | AUC | 0.829 | 0.829 | 0.880 | 0.863 | 0.821 | 0.538 |
| **Oligoastrocytoma**  **Ⅱ *vs* Ⅲ** | Cut-off | 84.25 | 118.5 | 9.200 | 20.99 | 193.6 | 24.40 |
|  | Sensitivity (%) | 100.0 | 100.0 | 100.0 | 77.78 | 100.0 | 77.78 |
|  | Specificity(%) | 100.0 | 100.0 | 83.33 | 83.33 | 66.67 | 66.67 |
|  | AUC | 1.000 | 1.000 | 0.907 | 0.722 | 0.852 | 0.602 |
| **GradeⅡ**  **Astrocytoma *vs* Oligodendroglioma** | Cut-off | 42.48 | 66.00 | 7.360 | 17.09 | 84.81 | 23.00 |
|  | Sensitivity (%) | 84.62 | 76.92 | 76.92 | 84.62 | 84.62 | 76.92 |
|  | Specificity(%) | 76.92 | 84.62 | 84.62 | 84.62 | 76.92 | 100.0 |
|  | AUC | 0.799 | 0.781 | 0.828 | 0.870 | 0.840 | 0.870 |
| **IDH^MUT^/1p/19q^+^**  **Ⅱ *vs* Ⅲ** | Cut-off | 84.25 | 118.5 | 9.110 | 20.99 | 193.6 | 53.80 |
|  | Sensitivity (%) | 93.75 | 93.75 | 93.75 | 87.50 | 100.0 | 87.50 |
|  | Specificity(%) | 100.0 | 100.0 | 81.82 | 81.82 | 63.64 | 45.45 |
|  | AUC | 0.977 | 0.983 | 0.920 | 0.852 | 0.864 | 0.568 |
| **IDH^MUT^/1p/19q^−^**  **Ⅱ *vs* Ⅲ** | Cut-off | 41.80 | 152.0 | 7.180 | 15.63 | 84.81 | 28.40 |
|  | Sensitivity (%) | 87.50 | 100.0 | 87.50 | 87.50 | 87.50 | 87.50 |
|  | Specificity(%) | 77.78 | 66.67 | 88.89 | 88.89 | 88.89 | 55.56 |
|  | AUC | 0.833 | 0.847 | 0.875 | 0.792 | 0.870 | 0.625 |
| **GradeⅡ**  **IDH^MUT^/1p/19q^+^ *vs* IDH^MUT^/1p/19q^−^** | Cut-off | 42.48 | 49.87 | 9.03 | 17.06 | 96.01 | 24.80 |
|  | Sensitivity (%) | 100.0 | 100.0 | 75.00 | 100.0 | 91.67 | 91.67 |
|  | Specificity(%) | 68.75 | 62.50 | 87.50 | 56.25 | 56.25 | 93.75 |
|  | AUC | 0.807 | 0.802 | 0.854 | 0.745 | 0.792 | 0.948 |
| **GradeⅡ**  **IDH^MUT^/1p/19q^+^ *vs* IDH^WT^** | Cut-off | 41.80 | 62.23 | 7.180 | 15.53 | 84.81 | 24.80 |
|  | Sensitivity (%) | 100.0 | 83.33 | 91.67 | 100.0 | 100.0 | 91.67 |
|  | Specificity(%) | 87.50 | 87.50 | 87.50 | 87.50 | 87.50 | 87.50 |
|  | AUC | 0.885 | 0.865 | 0.865 | 0.875 | 0.938 | 0.948 |

Note： IDH^MUT^/1p/19q^+^ IDH-mutant with 1p/19q-intact type, IDH^MUT^/1p/19q^−^ IDH-mutant with 1p/19q-codeleted type, IDH^WT^ IDH-wild type

Fig. S1


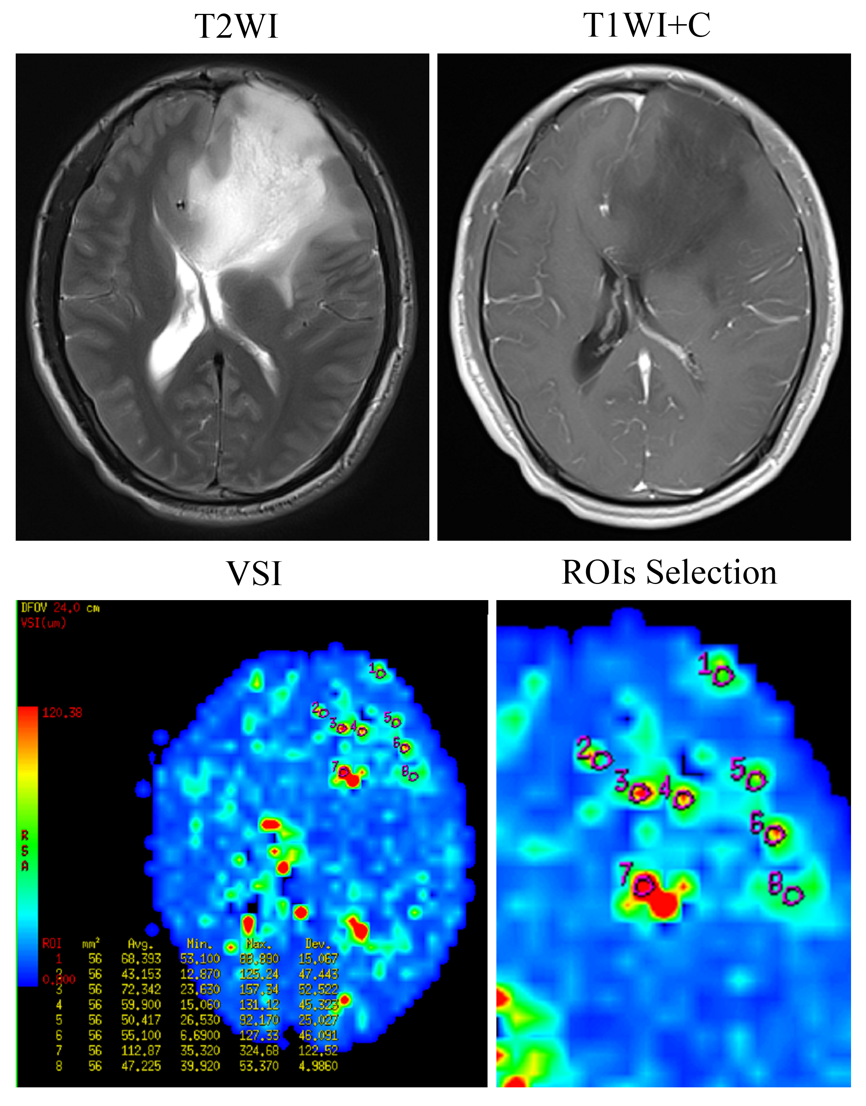


**Fig. S1** Vessel size index measurements. At least five circular regions of interests (ROIs) with the same size of 56mm^2^ were selected within the solid component region of the tumor by referencing conventional MR images.

Fig. S2


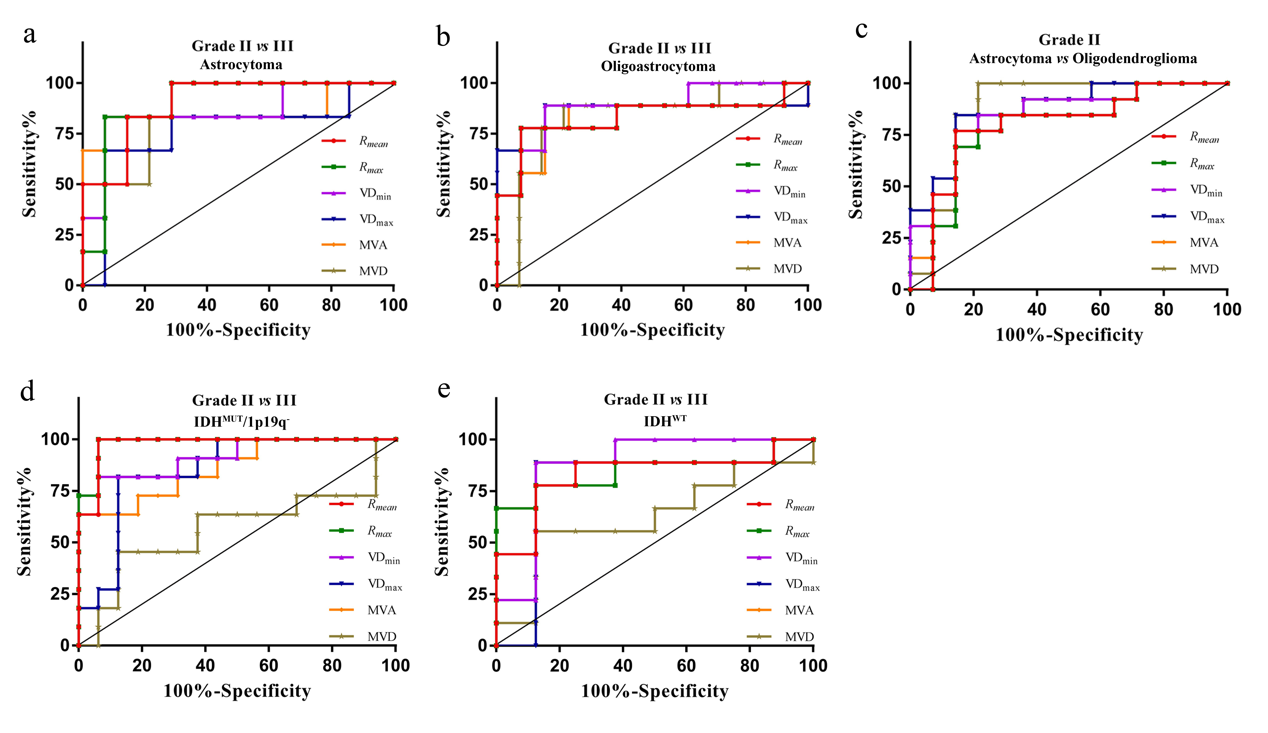


**Fig. S2** Receiver operating characteristic curves for differentiating grade II and grade III in astrocytoma (a), grade II and grade III in oligoastrocytoma (b), astrocytoma and oligodendroglioma in grade II (c), grade II and grade III in IDH-mutant with 1p/19q-codeletion (IDH^MUT^/1p/19q^−^) type (d), grade II and grade III in IDH-wild (IDH^WT^) type (e). *R_mean_* average vessel size index, *R_max_* maximum vessel size index, VD_min_ short vascular diameter, VD_max_ long vascular diameter, MVA microvascular area, MVD microvascular density

Fig. S3


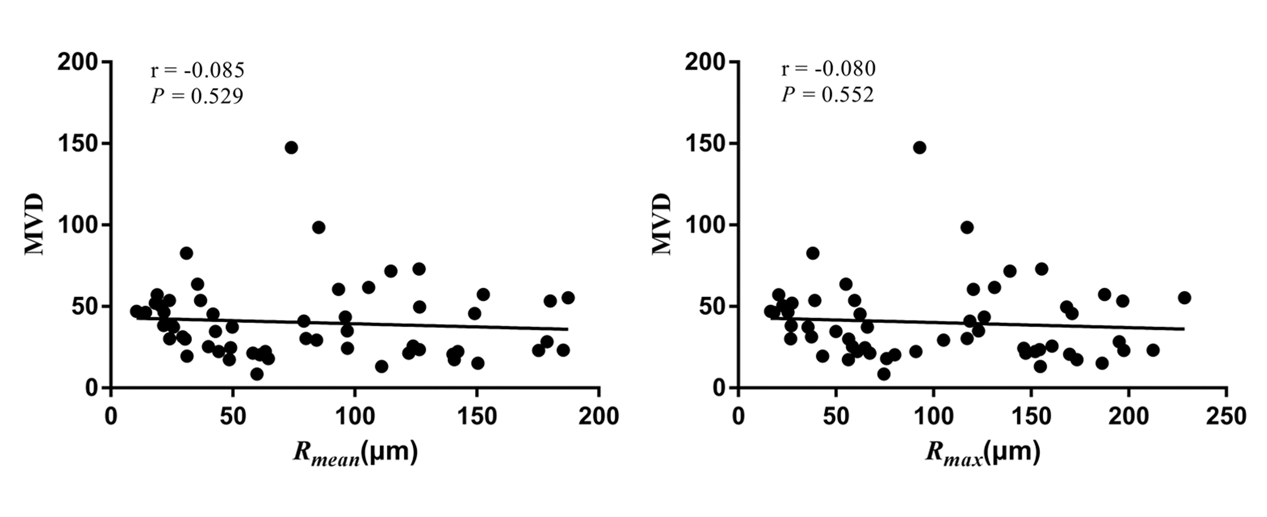


**Fig. S3** There was no correlation between microvascular density (MVD) and average vessel size index (*R_mean_*), *R_max_* respectively.
